# *QuickStats*: Unintentional Drowning* Death Rates^†^ of Children and Adolescents Aged 0–17 Years, by Sex and Age Group — United States, 2020–2021

**DOI:** 10.15585/mmwr.mm7234a7

**Published:** 2023-08-25

**Figure:**
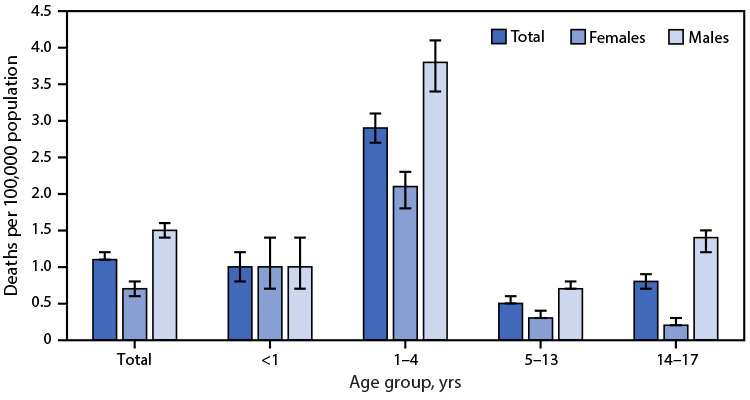
During 2020–2021, the unintentional drowning death rate was 1.1 deaths per 100,000 population among children and adolescents aged 0–17 years. Rates were higher among males (1.5) than females (0.7). Among children aged <1 year, boys and girls had similar unintentional drowning death rates (1.0), whereas rates were higher for males than for females among those aged 1–4 (3.8 versus 2.1), 5–13 (0.7 versus 0.3), and 14–17 years (1.4 versus 0.2). Rates were highest among those aged 1–4 years among all children and adolescents and among all males and females compared with other age groups.

**Source:** National Center for Health Statistics, National Vital Statistics System, Mortality Data, 2021. https://www.cdc.gov/nchs/nvss/deaths.htm

For more information on this topic, CDC recommends the following link: https://www.cdc.gov/drowning/index.html

